# Microspectroscopic Evidence of Cretaceous Bone Proteins

**DOI:** 10.1371/journal.pone.0019445

**Published:** 2011-04-29

**Authors:** Johan Lindgren, Per Uvdal, Anders Engdahl, Andrew H. Lee, Carl Alwmark, Karl-Erik Bergquist, Einar Nilsson, Peter Ekström, Magnus Rasmussen, Desirée A. Douglas, Michael J. Polcyn, Louis L. Jacobs

**Affiliations:** 1 Division of Geology, Department of Earth and Ecosystem Sciences, Lund University, Lund, Sweden; 2 MAX-lab, Lund University, Lund, Sweden; 3 Chemical Physics, Department of Chemistry, Lund University, Lund, Sweden; 4 Department of Anatomy, Midwestern University, Glendale, Arizona, United States of America; 5 Division of Organic Chemistry, Department of Chemistry, Lund University, Lund, Sweden; 6 Department of Biology, Lund University, Lund, Sweden; 7 Division of Infection Medicine, Department of Clinical Sciences, Lund University, Lund, Sweden; 8 Roy M. Huffington Department of Earth Sciences, Southern Methodist University, Dallas, Texas, United States of America; Paleontological Institute of Russian Academy of Science, United States of America

## Abstract

Low concentrations of the structural protein collagen have recently been reported in dinosaur fossils based primarily on mass spectrometric analyses of whole bone extracts. However, direct spectroscopic characterization of isolated fibrous bone tissues, a crucial test of hypotheses of biomolecular preservation over deep time, has not been performed. Here, we demonstrate that endogenous proteinaceous molecules are retained in a humerus from a Late Cretaceous mosasaur (an extinct giant marine lizard). In situ immunofluorescence of demineralized bone extracts shows reactivity to antibodies raised against type I collagen, and amino acid analyses of soluble proteins extracted from the bone exhibit a composition indicative of structural proteins or their breakdown products. These data are corroborated by synchrotron radiation-based infrared microspectroscopic studies demonstrating that amino acid containing matter is located in bone matrix fibrils that express imprints of the characteristic 67 nm *D*-periodicity typical of collagen. Moreover, the fibrils differ significantly in spectral signature from those of potential modern bacterial contaminants, such as biofilms and collagen-like proteins. Thus, the preservation of primary soft tissues and biomolecules is not limited to large-sized bones buried in fluvial sandstone environments, but also occurs in relatively small-sized skeletal elements deposited in marine sediments.

## Introduction

The fossil record is capable of exceptional preservation and occasionally labile and decay-prone tissues, such as skin and melanosomes (color-bearing organelles), are preserved as phosphatized remains or organic residues with a high degree of morphological fidelity [1], [2]. Yet, whether multimillion-year-old fossils harbor original organic components remains controversial [3], [4], and, if they do, a positive identification of these biomolecules is required.

Recent attempts to detect type I collagen (the main structural protein in skeletal tissues) in fossil bones have relied largely on liquid chromatography tandem mass spectrometry (LC/MS/MS) [5], [6], which provides identification of the peptide sequences but has the drawback of being based on whole bone extracts rather than location-specific tissues (the localization of the collagenous peptides has instead been inferred largely from epitope data [6]). Questions have also been raised concerning the authenticy of the amino acid sequences obtained from this form of analysis [3], and whether or not it is possible to distinguish between a few peptides derived from animal collagens and collagen-like proteins from, e.g., bacteria [4].

Here, we present the results from a broad array of biochemical and molecular analyses of fibrous bone tissues isolated from an exceptionally preserved 70 Ma mosasaur (a Cretaceous marine lizard [7]) humerus (IRSNB 1624; Institut Royal des Sciences Naturelles de Belgique) referred to the genus *Prognathodon* from the early Maastrichtian Ciply Phosphatic Chalk of Belgium. Specifically, we employ synchrotron radiation-based infrared microspectroscopy (IR) because this technique provides information on complex organic molecules in selected microstructures [8], [9].

## Methods

### Osteohistological preparation

An approximately 5 mm thick section of the diaphysis was extracted from IRSNB 1624 using a sterilized diamond saw. Thereafter, the sample was vacuum embedded in polyester resin to prevent shattering during slide preparation. Once embedded, two approximately 1 mm thick cross-sections were cut from the block. Each section was attached to a petrographic slide with polyester resin and ground to optical translucency. The cross-sections were imaged using a Minolta Dynax 505si camera with an AF 100 macrolens. The sample embedded in polyester resin was not used for biochemical and molecular analyses.

### Initial preparation of samples used in biochemical and molecular analyses

Prior to analyses, IRSNB 1624 was cleaned extensively by abrasion using sterile instruments and gloves to remove any bone material that could have been contaminated with exogenous biomolecules from either adhering sediments or post-excavation treatment. In order to isolate organic microstructures, small samples were extracted from the mid-shaft of the element using a sterilized diamond saw. These samples were segregated from that to be embedded in polyester resin (see above), and were demineralized using ethylenediaminetetra-acetic acid (EDTA, 5.5%, pH 8.0) for 6–11 days, with daily buffer changes (see [10] for details). The residues were rinsed in phosphate-buffered saline (PBS, pH 6.5) and washed multiple times in Epure water. Selected samples were fixed in 4% paraformaldehyde, whereas others remained unfixed when studied. The tissues were photodocumented using a Nikon Coolpix 990 camera attached to a binocular microscope (OM). All tissue samples were placed in sealable, sterile containers and kept refrigerated at 4°C until examined (usually within hours or days).

### Scanning electron microscopy (SEM)

Bone tissues from IRSNB 1624 were collected on glass discs and either sputter-coated with an 80/20 mixture of gold and palladium or studied uncoated under low vacuum using a Hitachi S-3400N scanning electron microscope. Energy dispersive X-ray analysis (EDAX) was used to establish the chemical composition of selected microstructures.

### Transmission electron microscopy (TEM)

Following demineralization, samples from IRSNB 1624 and an extant monitor lizard (*Varanus exanthematicus*, LO 10298; Department of Earth and Ecosystem Sciences, Lund University) were prefixed overnight with 2.5% glutaraldehyde in PBS and then postfixed with 1% OsO_4_. Thereafter, the samples were stepwise dehydrated with 50%, 70%, 96% and finally pure alcohol. The alcohol was replaced with acetone, which in turn was stepwise substituted with epoxy resin, thereby embedding the tissues. The epoxy was left to polymerize at 60°C for 48 h. Embedded samples were first trimmed with a razor blade and then 2 µm thick sections were cut using a glass knife mounted on an ultrotome. Cut sections were stained with Azur-2 methylene blue and examined under a light microscope for a general overview. For the ultra-thin sectioning a diamond knife was used, cutting 50–100 nm slices that were applied to copper grids. The grids were treated with lead citrate and uranyl acetate for increased contrast and then inserted in a JEOL JEM-1230 transmission electron microscope. The microscope ran at 80 KeV and sites of interest were photographed using a MultiScan 701 camera.

### Acid etching

Untreated portions of the inner cortex of IRSNB 1624 were cut into small slabs using a sterilized diamond saw. Freshly exposed surfaces were polished, acid etched with 37% phosphoric acid for 6–23 s, washed with 5% sodium hypochlorite for 5 min, rinsed with Epure water, air dried at room temperature, sputter-coated with an 80/20 mixture of gold and palladium, and finally examined using a Hitachi S-3400N scanning electron microscope (see [11] for details).

### Staining with Aniline blue

Demineralized samples were stepwise dehydrated with 70%, 96% and finally pure alcohol. The alcohol was replaced with xylene, which in turn was substituted with a mixture of xylene and melted paraffin, and finally pure paraffin. The paraffin was left to harden for 24 h at 56°C. The prepared samples were then embedded in paraffin blocks, and left to air dry at room temperature for 30 min. The embedded samples were cut into 10 µm thick sections using a Leitz rotary microtome and placed on glass slides. The paraffin was removed in a stepwise procedure using xylene, 100%, 96% and 70% alcohol, respectively. Next, the slides were rinsed with Epure water and treated with 5% phosphorus-wolfram acid for 1.5 h, washed in Epure water, and stained with Aniline blue dye for 1.5 h. Stained tissues were imaged under a binocular microscope using a Nikon Coolpix 990 camera.

### Confocal laser scanning microscopy

Demineralized bone samples were stained with either 0.05 µM DAPI (4′–6′-diamidino-2-phenylindole) overnight, or propidium iodide diluted 1∶1000 (w/v) for 2 h, both in PBS at 4°C. Bone tissue samples were mounted in a staining solution with 0.17 mm thick coverslips. The specimens were examined with a Zeiss LSM 510 META confocal laser scanning microscope using a 405 nm LED laser for DAPI excitation and a 561 nm LED laser for propidium iodide excitation. Specimens were scanned at Nyquist optimal frequency using either a ×40/1.3 oil immersion Plan-Neofluar objective or a ×63/1.4 oil immersion plan apochromate objective. Projections of stacks of confocal images were obtained with the LSM 510 software, and exported as uncompressed TIFF files for formatting in Adobe Photoshop.

### Immunofluorescence

Small tissue samples were demineralized in EDTA, rinsed several times in Epure water, and then fixed overnight at 8°C in 4% paraformaldehyde in 0.1 M Sörensen's phosphate buffer (pH 7.2). The samples were then rinsed in 0.1 M PBS, and cryoprotected by infiltration with 25% sucrose in PBS. Thereafter, the samples were sectioned in a cryostat (Microm HM560), and 10 µm thick sections were collected on chrome alum/gelatin-coated slides, and left to air dry. The sections were stored in the dark at 8°C until being further processed.

For immunofluorescence, the slides were rinsed twice in PBS, followed by a rinse in PBS containing 0.25% Triton X-100 (PBS-TX). The sections were then incubated overnight in a dark humidity chamber at room temperature, with rabbit anti-collagen (chicken) type I (US Biological C7150-13B) diluted at 1∶40 or 1∶80 in PBX-TX containing 1% bovine serum albumin. After several rinses in PBS, the sections were incubated for 1 h in a dark humidity chamber at room temperature with goat anti-rabbit IgG conjugated to Alexa488 (Molecular Probes/Invitrogen) diluted at 1∶200 in PBS containing 1% bovine serum albumin. The sections were then rinsed several times in PBS, and cover-slipped with FluoroSave as mounting medium.

The sections were examined with a Zeiss LSM 510 META confocal microscope using a ×63/1.4 oil immersion plan apochromate objective, 488 nm (argon ion laser) as excitation light, and a detection of 505–530 nm.

### Amino acid analysis

The amino acid analyses were made by ion exchange chromatography performed at the Amino Acid Analysis Centre, Department of Biochemistry & Organic Chemistry, University of Uppsala, Uppsala, Sweden.

### Infrared microspectroscopy

Demineralized bone samples from IRSNB 1624 were washed multiple times in Epure water. The solutions were then placed on CaF_2_ spectrophotometric plates and air dried under a hood at room temperature. After the solvent had evaporated, the samples were examined uncoated at low vacuum using a light microscope and/or a Hitachi S-3400N scanning electron microscope. Isolated fibrous tissues were photographed for subsequent identification under infrared microspectroscopy.

For comparison, demineralized bone samples from a humerus of an extant monitor lizard (LO 10298) were partly digested with 5 mg/ml collagenase enzyme in 25 mM Tris, 5 mM CaCl_2_ for three days at room temperature to release fibers, osteocytes and vessels. The residues were suspended in Epure water and rinsed multiple times, and then casted onto CaF_2_ spectrophotometric plates for initial identification under SEM.

Infrared microspectroscopy was performed at beamline 73, MAX-lab, Lund University. The infrared microscope combines a Hyperion 3000 and a Bruker IFS66/v FTIR spectrometer. A single element MCT with a 100×100 µm detector element was used for all measurements. The microscope was operated in transmission mode using apertures that ranged from 10×10 to 140×140 µm. Only for the largest aperture was a confocal arrangement used. The smallest aperture was used with synchrotron radiation as the light source. All other samples were measured with a conventional light source. A ×15 objective and a condenser were employed for all measurements. This arrangement gives a visible magnification of ×215 for the video camera in the microscope, which was used to locate relevant structures in the samples. Fibrous tissues were localized from OM or SEM photographs. As a substrate, 2 mm thick CaF_2_ spectrophotometric plates were used, and a clean CaF_2_ plate was used as reference.

In order to link the OM and SEM observations firmly to the microspectroscopic data a number of criteria had to be fulfilled. Firstly, the spectral measurements should be recorded well inside the boundaries of the fibrous tissues. Secondly, the area recorded should exhibit transmission throughout the spectral region. Because the number of structures identified under OM or SEM was limited and since we cannot control the thickness of the generally small-sized fibrous tissues, the higher spatial resolution and flexibility when using high-brilliance synchrotron infrared light was necessary. Accordingly, a 10×10 µm aperture was employed when synchrotron infrared light was used. In order to obtain a similar signal under conventional light, an aperture of at least 140×140 µm was necessary (i.e., the measured area was 196 times larger than that measured with synchrotron infrared light).

The homogeneity of the mosasaur samples was investigated by measuring several different regions within the fibrous tissues identified under OM or SEM. In total twenty arbitrary regions were measured in each sample and compared for homogeneity. This procedure was performed in order to get good representation of the sample and to minimize contributions from inhomogeneities and artifacts. Potential variations within original spectra as well as variations within sets of arbitrary regions were examined manually. The resulting spectra for different sets were similar to each other, thereby demonstrating the homogeneity both within individual fiber bundles and between fibrous tissues from different regions.

### Microstructures, biomolecules, cells, and biofilms examined

- Isolated fibrous tissues from IRSNB 1624 (*Prognathodon*) – multiple samples.
- Isolated osteoid from the humerus of LO 10298 (*Varanus exanthematicus*) – multiple samples.
- Bovine type I collagen (Fisher Scientific).
- *Enterococcus faecalis* biofilm. A clinical isolate of *E*. *faecalis* was grown in two 200 µl wells of polystyrene plastic in tryptic soy broth supplemented with 0.25% glucose (TSBG, DIFCO) for 24 h. The resulting biofilm was washed once in PBS and resuspended in 50 µl of the same buffer.
- *E. faecalis* planctonic cells. The same isolate was cultivated for 18 h in TSBG and bacteria from 400 µl solution were pelleted and resuspended in 50 µl of PBS.
- *Propionibacterium acnes* biofilm. A clinical isolate of *P*. *acnes* (AD49) was grown in two 200 µl wells of polystyrene plastic as described in [12]. The resulting biofilm was washed once in PBS and resuspended in 50 µl of PBS.
- *P. acnes* planctonic cells. AD49 bacteria were cultivated as described in [12] and bacteria from 400 µl solution were pelleted and resuspended in 50 µl of PBS.
- SclB, a collagen-like protein derived from *Streptococcus pyogenes*, was expressed as a fusion with Glutathione S-transferase (GST). The protein was purified as described in [13] followed by buffer exchange to PBS using Zeba desalt spin columns (Pierce).
- GST in PBS (GST was purified as described for GST-SclB). GST and SclB each contains about 200–300 amino acids, and the spectra obtained from pure GST were rather like those representing the GST-SclB fusion. This is because infrared microspectroscopy probes the local molecular structure, and consequently differentiation between long and short amino acid chains is not efficiently documented by this technique. Nonetheless, multiple sites of the GST-SclB sample were measured with similar results to suggest that, at least locally, the secondary structure of GST and SclB is comparable.
- Phosphate-buffered saline (PBS). Because all microbial structures examined herein were suspended in PBS, a sample of this buffer solution was measured to ensure that the contribution from it to the infrared spectra was small. It was found that although the signal from PBS cannot be neglected, all spectra are clearly dominated by genuine microbial structures.
- Negative controls. In order to exclude the possibility that we were analyzing contaminants introduced during the pre-treatment process, such as EDTA, collagenase and/or keratins, we examined pure EDTA and additional fossil bones from a number of localities that were prepared in exactly the same way as were IRSNB 1624 and our monitor lizard samples. However, with the exception of pure EDTA (which is washed away during the rinsing process of our samples, given the lack of amide band-containing matter in the ‘background’), we were unable to detect any amino acid-containing matter in the control samples (i.e., we did not see any amide bands, although the fossil tissues occasionally did contain biomolecules), to suggest that neither EDTA, collagenase, nor keratins did contribute in any significant way to the spectra recorded for IRSNB 1624, LO 10298 and the microbial structures.

### Mass spectrometer analysis

With minor modifications, total lipid extracts were collected following the extraction procedure as described in [14]. Mass spectrometry was performed on lipid extracts from the inner part of the cortex. Small bone samples were ground in a sterilized agar mortar and subject to lipid extraction in 2×1 ml CH_2_Cl_2_:MeOH (4∶1). The samples were filtrated through a freshly prepared glass wool-filter and the solvent was removed through evaporation. Two mg of each sample was dissolved in 100 µl CH_2_Cl_2_ and subject to gas chromatography-mass spectrometry. The entire extraction procedure, using the same batches of solvent and equivalent laboratory equipment, was then repeated in order to produce blank samples. The blanks were compared with the initial samples using proton nuclear magnetic resonance spectroscopy, and were found to contain no detectable amounts of triterpenes or other organic molecules apart from the solvent and a small residue of saturated hydrocarbons.

Mass spectrometry was carried out using a Walters GCT controlled by Lynx software and equipped with a HP-5 column (30 m, 0.32 mm I.D., 0.25 µm film). The temperature was held at 70°C for 1.7 min. It was then increased by 5°C/min until 280°C was reached and kept so throughout the remainder of the analysis. Ion source temperature was 250°C and the ionizing energy was 70 eV.

### ^14^C analysis

In order to remove absorbed carbonates and humic acids, a small bone sample (2 g) was pre-treated according to the acid-alkali-acid method; i.e., it was washed in 2% HCl solution at 80°C for 12 h, then in 5% NaOH solution at 80°C for 5 h, followed by a final wash in 2% HCl. After this procedure, the dried residues (258 mg) were combusted to CO_2_ using CuO as oxidizing agent. The CO_2_ was then mixed with H_2_ gas and reduced to elemental carbon before being analyzed at Lund University Radiocarbon Dating Laboratory using single stage accelerator mass spectrometry. Approximately 5 mg of carbon was produced, of which 3 mg was used in the analysis.

### DNA extraction and PCR

One gram of cortical bone was crushed to a coarse powder with a sterile pestle and mortar. A lipid extraction was performed under a fume hood using methylene chloride and methanol (4∶1). The lipid layer was discarded. The DNA extraction was performed in separate laboratories to where the polymerase chain reactions (PCRs) were carried out, and separate lab coats were worn. Gloves were changed frequently. Lab equipment and reagents were UV radiated and all lab areas and surfaces wiped regularly with bleach or HCl. Because the chaotropic agent GuSCN was used, most of the extraction was carried out under a fume hood. A variation of the silica extraction method [15], [16] was used. Silica extraction was found to reduce the amount of PCR inhibitors present [17]. An additional extraction step was included whereby NaH_2_PO_4_ was added to the bone powder to break down hydroxyapatite to which DNA is adsorbed. A blank extraction was also performed in parallel to the mosasaur DNA extraction. The amount of extracted DNA was determined using a spectrophotometer.

Conserved lepidosaurian PCR primers were designed from conserved regions within the 12S and 16S ribosomal RNA mitochondrial genes. It was suspected that any DNA present in the bone would be exceedingly degraded so only the amplification of very short fragments of 20 to 60 base pairs was attempted. Any pre-PCR work was carried out in the same lab as the DNA extraction. Two negative controls – one with no DNA and one using the blank extract – were used, along with a positive control using modern squamate DNA. Amplification was carried out using AmpliTaq Gold (Roche). PCR products were ligated into pGEM-T Easy Vector (Promega) and cloned. As many colonies as possible were picked for colony PCR and the resulting PCR products were then sequenced. BLAST searches determined whether the clone sequences were possibly authentic mosasaur DNA or contamination.

## Results

### Combined analyses of IRSNB 1624

**Figure 1:**
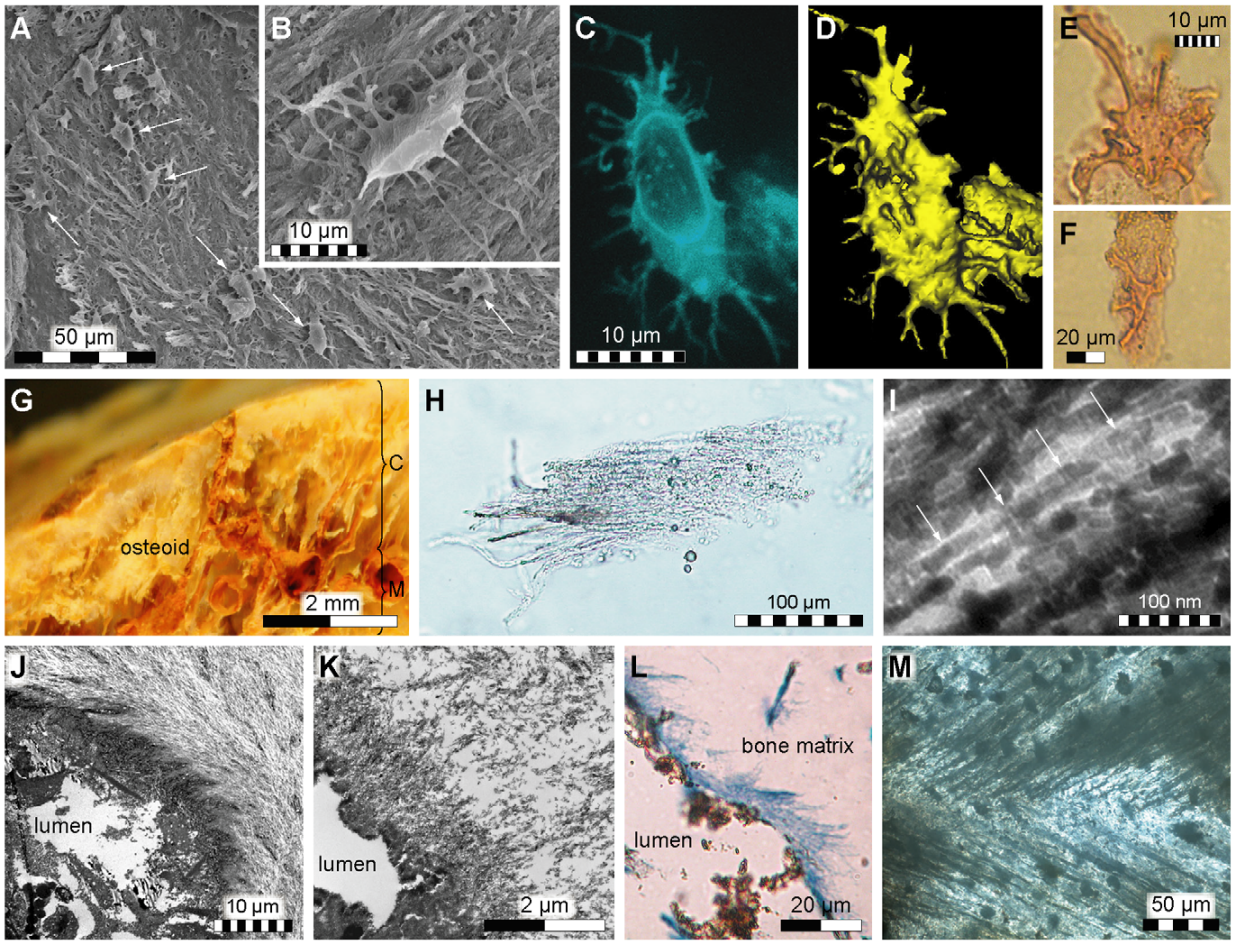
Fibrous tissues and microstructures recovered from IRSNB 1624. (A) Secondary electron micrograph of acid etched cortical bone showing fibrous tissues and what appears to be part of the osteocyte-lacunocanalicular system (osteocyte-like entities at arrows). (B) Osteocyte-like structure in lacuna within fibrous tissues. (C) Isolated osteocyte-like form visualized with fluorescent dye. (D) Topographic image of the same specimen as in C to illustrate the three-dimensional arrangement of the presumed cytoplasmic protrusions. (E, F) Light micrographs of fossil microstructures that are consistent in size and morphology to osteocytes or pericytes enfolding the outer surface of two vessel fragments. (G) Light micrograph of demineralized mosasaur bone tissues showing possible remains of the osteoid associated with vessel-like structures (C = cortex, M = medulla). (H) Light micrograph of an isolated fiber bundle. (I) TEM-image of demineralized mosasaur bone showing parallel-oriented fibrils. The spacing of the arrows indicates a 67 nm axial repeat *D*-banding pattern, which in modern bone is characteristic of collagen. (J) Transverse section (TEM-image) of a blood vessel from cortical bone of an extant monitor lizard humerus (LO 10298). Note the hair-like bone matrix fibers that are coiled around the canal wall. (K) Corresponding structures in the mosasaur humerus. (L) Histochemical staining of demineralized mosasaur bone suggesting the presence of connective tissue (blue) in the hair-like fibers that line a partially ruptured canal wall (the fracturing presumably occurred during the preparation of the sample). (M) Light micrograph (thin section) of untreated mosasaur bone showing fibers embedded in hydroxyapatite.

Prior to examination IRSNB 1624 was almost complete and intact. A subsequent histological analysis revealed well-preserved microstructural features and no evidence of recrystallization. Demineralization of the diaphysis liberated numerous free-floating, vessel-like structures joined in a discrete network with a predominantly radial orientation, similar to those previously reported in dinosaurs [6], [10]. Associated with the vessel-like forms were cell-like features (Figure 1A–F) and a fibrous substance that, in modern bone, would represent the organic phase of the extracellular matrix; i.e., the osteoid (Figure 1G). The fibrous organization of the organic matter was demonstrated by optical and scanning electron microscopy (Figures 1A, B and H). Furthermore, application of a standard histochemical dye (Aniline blue) revealed that the fiber-like structures take up stain as does recent connective tissue (Figure 1L). Under transmission electron microscopy, regions of well-aligned, cross-striated fibril-like structures with a faint 67 nm banding periodicity were seen (Figure 1I, arrows). Fiber-like forms with a similar typical axial periodicity were also found coiled obliquely around some canal walls (Figure 1K) and are, in modern animals, comprised primarily of mineralized fibrillar collagens (Figure 1J) [18].

**Figure 2:**
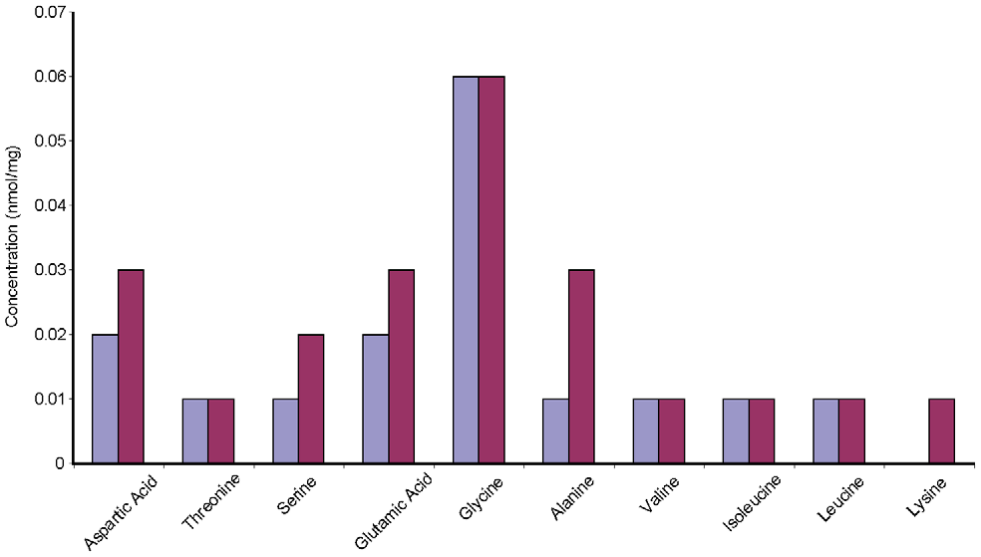
Amino acid content of IRSNB 1624 (two batches). The majority of the amino acids present are aspartic acid, serine, glutamic acid, glycine, and alanine. Together, these amino acids make up approximately 60% of the residues in modern collagen [19]. Additionally, the molecular composition of collagen incorporates glycine at every helical turn, resulting in a high concentration of this amino acid [19], [35]. Low resolution and co-extraction contaminants prevented analysis of amino acids in the 440 nm region (such as proline and hydroxyproline).

To test the possibility of endogenous macromolecular preservation, amino acid analyses were performed on soluble extracts of IRSNB 1624. The amino acid profiles we obtained have a composition potentially indicative of fibrous structural proteins (Figure 2), such as collagen [19], suggesting that the proteinaceous molecules isolated from IRSNB 1624 may contain this protein or its degradation products.

**Figure 3:**
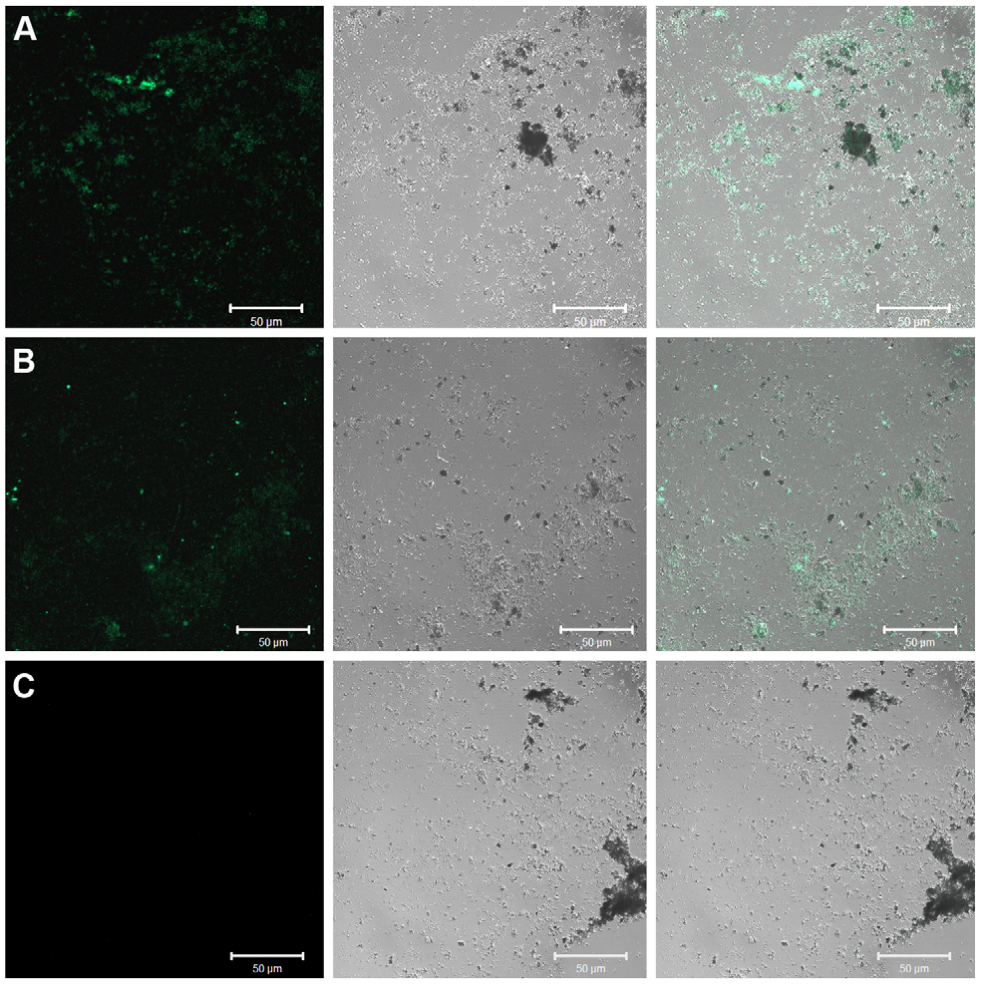
In situ immunofluorescence of demineralized bone extracts of IRSNB 1624. (A) With antibodies to type I collagen (diluted 1∶40). (B) With antibodies to type I collagen (diluted 1∶80). (C) No primary antibodies added (negative control). Although somewhat fragmented during the pre-treatment process, the samples presented in A and B still show reactivity against antibodies raised against type I collagen. The samples are illustrated as confocal section of immunofluorescence (left), Nomarski differential interference contrast, DIC (center), and combined immunofluorescence and DIC (right).

**Figure 4:**
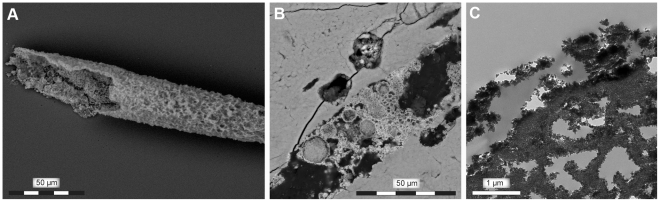
Vessel-like structures obtained from IRSNB 1624. (A) Scanning electron micrograph of an isolated vessel-like structure comprised primarily of iron oxide. (B) Scanning electron micrograph of iron oxide crystals and oxidized pyrite framboids within a longitudinally sectioned vascular canal. (C) Transmission electron micrograph of iron oxide crystals lining the inside of a vessel wall.

**Figure 5:**
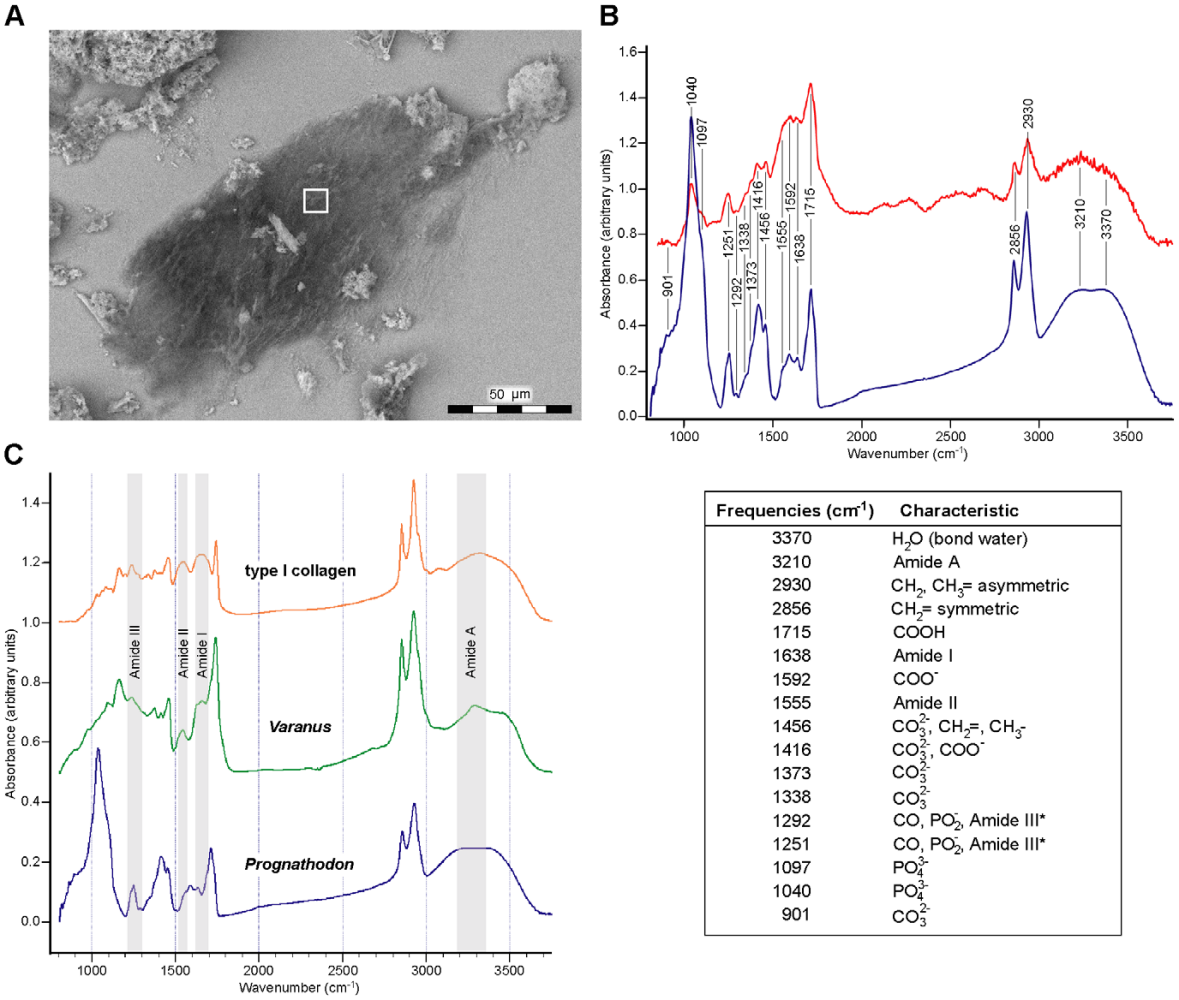
Infrared spectra of mosasaur and *Varanus* fibrous tissues together with type I collagen. (A) SEM-image of a partly demineralized fiber bundle isolated from IRSNB 1624 (white 10×10 µm square indicates measured area). (B) Synchrotron infrared spectrum (red) from the fiber bundle depicted in A together with a spectrum (blue) recorded with a 140×140 µm aperture (conventional light source) showing e.g., typical amide band frequencies and peaks attributed to phosphate and carbonate bands. Spectra from five arbitrary spatial regions were combined to produce the blue spectrum, and frequencies in the 1500–1750 cm^−1^ interval derive from the peak fit analysis presented in Figure 7A. *Uncertain assignment (see main text). (C) Infrared spectra of mosasaur and monitor lizard (LO 10298) tissues together with the compound signature for type I collagen. Note extreme similarities in the peak positions between the three spectra, and characteristic absorption peaks for the amide bands I–III and A. Spectra from five different regions were co-added to produce the type I collagen and monitor lizard spectra.

In order to identify potentially protein-harboring tissues, demineralized bone samples from IRSNB 1624 were examined using in situ immunofluorescence, whereby regions showing reactivity to antibodies raised against type I collagen were observed (Figure 3). Additional samples were subject to infrared microspectroscopy. Whereas the vessel-like forms primarily comprised iron oxide (Figure 4), the infrared absorbance spectra of the associated fiber bundles (Figure 5A) exhibited peaks corresponding to proteinaceous materials (Figure 5B). Typical amide bands [8], [20] were found at frequencies centered around 3210 (Amide A), 1635 (Amide I) and 1550 (Amide II) cm^−1^, respectively. Additionally, peaks in the 1250–1290 cm^−1^ region may represent Amide III [21], [22], although these bands are presumably dominated by C–O compounds and phosphate groups [23]. Amide bands are associated with stretching and bending vibrations of the peptide (CO–NH) bonds, and their positions and intensities are sensitive to structural changes of the conformation of the protein molecule [8], [24]. Vibrational bands corresponding to nonproteinaceous material were also indentified (Figure 5B), and ascribed to, among other things, phosphate groups (1040–1100 and 1250–1290 cm^−1^) and A-B carbonate (900, 1335–1375 and 1410–1460 cm^−1^) of hydroxyapatite [23]. From the prominent phosphate peak at 1040 cm^−1^ in Figure 5B (blue spectrum), it is obvious that IRSNB 1624 did not completely demineralize after incubation in EDTA, a characteristic confirmed by TEM studies, in which regions with multiple, stacked hydroxyapatite crystals were observed.

Because we investigated extremely small amounts of exceptionally rare and unique tissues, we prepared our samples with a minimum of pre-treatment in order to minimize potential deterioration of the biomolecules. Importantly, this approach did not isolate any particular molecules from the osteoid-like substance, but instead our IR data represent the sum of the contributions gathered from all biomolecules in the fibrous tissues. Thus, to facilitate comparisons with a relevant modern reference, bone tissue samples from an extant monitor lizard (LO 10298) were prepared in the same way as the mosasaur tissues. Additionally, because type I collagen is the most common protein in bone tissues [18], a vibrational spectrum was obtained for this protein as well. As shown in Figure 5C there are significant similarities between the three spectra to suggest a comparable molecular composition and a correlation of the vibrational frequencies of the component bonds.

**Figure 6 pone-0019445-g006:**
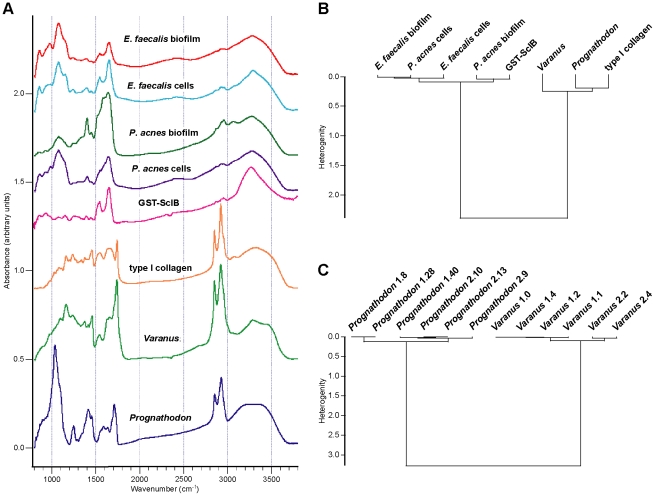
Infrared spectra of mosasaur and *Varanus* fibrous tissues, collagen and various microbial structures. (A) Absorbance spectra of biofilms of *Enterococcus faecalis* and *Propionibacterium acnes*, isolated cells of the two bacteria, a collagen-like protein produced by *Streptococcus pyogenes* (SclB, as a fusion to GST), type I collagen, and fibrous tissues of *Prognathodon* (IRSNB 1624) and *Varanus* (LO 10298). Spectra from four different regions were co-added to produce the spectra for the microbial biofilms and planktonic cells (note, however, that the spectrum for *P*. *acnes* cells only includes three co-added spectra). The GST-ScIB spectrum consists of nine co-added spectra. Different numbers of spectra were used to obtain comparable signal-to-noise ratios. (B) Cluster analysis based on the spectral regions 1200–1800 and 2785–3730 cm^−1^ (peptide bond and lipid interval, respectively). Note that this is not a phylogeny. (C) Cluster analysis based on six arbitrary spatial regions from two samples of *Prognathodon* fibrous tissues (all having the same spectral resolution, 4 cm^−1^, and a similar signal-to-noise ratio) and six arbitrary spatial regions from two samples of *Varanus* osteoid.

As a first step to test our findings, spectral comparisons were made with two bacterial biofilms (i.e., 3-dimensional aggregations of bacteria within a cohesive exopolysaccharide matrix [25]) and with a bacterial collagen-like protein [13] to assess the possibility of protein contamination from modern microbial sources. The vibrational spectra for the biofilms and bacterial protein are presented along with spectra for type I collagen and the mosasaur and monitor lizard in Figure 6A. The biofilms and collagen-like protein have compound signatures that are markedly different from those of the animal tissues and protein, reflecting disparity in molecular conformation and discrepancies in the vibrational modes of the component bonds.

This observation was further corroborated by cluster analysis of the spectral regions 1200–1800 and 2785–3730 cm^−1^, corresponding to the Amide I–III and lipid intervals, respectively, with Ward's algorithm [26]. All spectra were pre-processed using first derivative and vector normalization, and the spectral distances were calculated using the factorization method (Bruker OPUS 6.5 software). In Figure 6B two clusters are clearly distinguished and the mosasaur sample groups robustly with modern animal collagen and osteoid.

During our IR studies, we exploited the high-brilliance of synchrotron radiation light, allowing for a small aperture (10×10 µm), in order to record the spectra well within selected microstructures (Figure 5A). Additionally, a larger aperture (140×140 µm), allowing for a conventional light source, was utilized in order to establish spectral signatures at several locations and in different regions of the samples (Figure 5B, blue spectrum). The relative insensitivity of the aperture size with respect to the vibrational spectra is clearly seen in Figure 5B. An increase of the aperture resulted in some intensity redistribution while the frequencies remained largely unchanged. The integrity of the spectral signatures from the mosasaur fibrous tissues was tested with cluster analysis (as above) and compared with osteoid samples from a monitor lizard (LO 10298). Spectra from different spatial regions originating from two mosasaur samples and two *Varanus* osteoid samples were used. Consistently, the resulting dendrograms put the mosasaur samples in one grouping and the *Varanus* samples in another grouping (Figure 6C).

**Figure 7:**
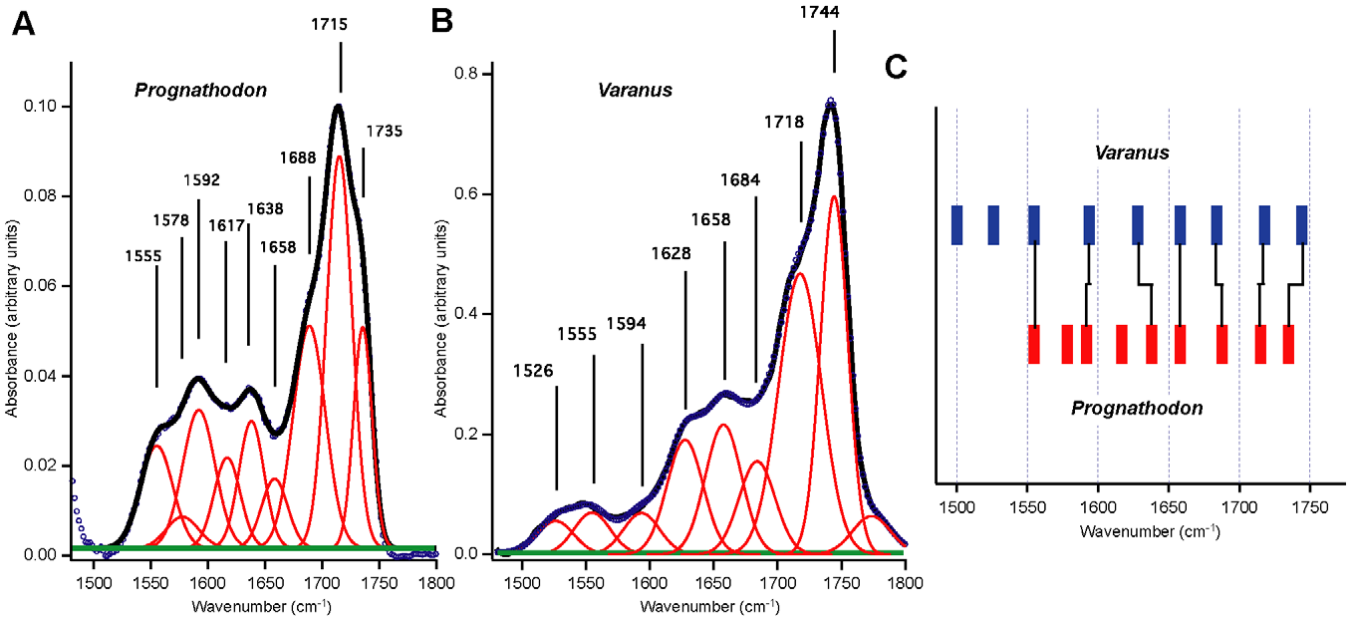
Peak fit analysis of mosasaur and monitor lizard IR spectra. (A) Peak fit analysis of the blue *Prognathodon* spectrum presented in Figures 5B, C and 6A. (B) Peak fit analysis of the *Varanus* spectrum shown in Figures 5C and 6A. The background of both spectra was subtracted using the Opus Concave rubber band correction method found in the Bruker OPUS 6.5 software package. Default values were used; i.e., 10 iterations and 64 baseline points. This correction marginally influenced peak fitting, positions and intensities, compared to those of a simple straight line subtraction, indicating a well-behaved and stable background. (C) Correlation diagram for frequencies obtained in the peak fit analyses of the *Prognathodon* and *Varanus* spectra.

Moreover, the quality of the mosasaur and monitor lizard spectra justified a preliminary peak fit analysis in the 1480–1800 cm^−1^ region (using IgorPro 6.05A), demonstrating that both spectra exhibit features characteristic of structural proteins (e.g., a triplet at approximately 1630, 1660 and 1690 cm^−1^, respectively [27]). We employed the minimal approach method while acknowledging the problems with peak fitting (e.g., by using an increased number of peaks an improved fit is obtained; however, at the same time the integrity of the individual peaks is reduced). From visual inspection of the background-subtracted mosasaur spectrum it was obvious that a minimum of six peaks was necessary in order to get a realistic agreement with the experimental spectrum. Additionally, in order to obtain reasonable line widths, i.e., ∼30 cm^−1^, three additional peaks were inserted. The resulting fit is shown in Figure 7A; the full widths at half maximum (FWHM) of the nine peaks are, on average, 28.7 cm^−1^ and all peaks are between 19 and 33 cm^−1^. Importantly, the three additional peaks at 1555, 1617 and 1658 cm^−1^ resulted in insignificant shifts (≤2 cm^−1^) of the six peaks in the original minimum fit. The same approach was then applied to the *Varanus* osteoid spectrum, resulting in a minimum of six peaks. By adding three more peaks at 1526, 1555 and 1594 cm^−1^, respectively, the line widths were reduced to an average FWHM of 29.4 cm^−1^, ranging between 25 and 40 cm^−1^ (Figure 7B). Furthermore, as seen in Figure 7C, there is a good correlation between the frequencies of the fitted peaks of the two spectra presented in Figure 7A and B.

A detailed analysis of the cross-links in collagen is presented in [27], particularly with respect to the 1630, 1660 and 1690 cm^−1^ bands. The *Varanus* spectrum showed a good correspondence with the intensity relation between these bands, as expected in any modern sample. In [27] the intensity distribution is 26% (1630 cm^−1^), 45% (1660 cm^−1^) and 29% (1690 cm^−1^), respectively, whereas the corresponding numbers in *Varanus* are 34% (1628 cm^−1^), 39% (1658 cm^−1^) and 27% (1684 cm^−1^), respectively. The mosasaur spectrum showed, as expected, a rather different intensity distribution: 26% (1638 cm^−1^), 15% (1658 cm^−1^) and 59% (1688 cm^−1^). Interestingly, a similar redistribution of intensities was observed when a sample of model pyridinoline cross-linked peptides was irradiated with UV light, resulting in degradation of the peptide chains through pyridinium ring cleavage [27]. In that experiment, the 1690 cm^−1^ band increased at the expense of the 1660 cm^−1^ band. In addition, a new band was growing at 1737 cm^−1^; both of these observations are consistent with our mosasaur spectrum (Figure 7A). Moreover, during the degradation of the modern peptide sample the separation (dip) observed between the bands in the 1550–1660 cm^−1^ region was smeared out (or filled) as a result of increased intensity between these bands. Again, this feature is consistent with our mosasaur spectrum (Figure 7A).

The high signal-to-noise ratio of the mosasaur spectra recorded with a large aperture and conventional light allowed examination of other possible degradational modifications of the fiber molecules. These alterations may reflect the loss of structural hierarchy through the unfolding of the protein molecules into a less ordered random coil form [8], [24], and fragmentation of the polypeptide chains [28], [29]. Oxidative deamination (a common alteration in sub-fossil collagen [19]) is capable of cleaving the N–C covalent bonds that link neighboring amino acids to one another [30], thereby forming C = O and C–O compounds (primarily carboxylic acid and carboxylate), which absorb in the 1250–1320, 1400–1460, 1560–1610, and 1670–1720 cm^−1^ regions [8], [20]. Studies of modern and sub-fossil collagens have shown that degradation causes an increase in absorbance of these bands [8], [28], [29]. Thus, extensive oxidative deamination may partially explain the unusually high peaks at 1416, 1592 and 1715 cm^−1^ in Figure 5B, as well as the broad and complex Amide I and II contours. Additionally, because the bones of extant marine tetrapods may exceed 60% lipid by weight [31], endogenous bone lipids presumably contribute to the intensity of the bands centered around 1400–1460, 1590, 1710–1740, and 2860–2930 cm^−1^ (Figure 5B).

### Rationale for excluding fungal growth and animal glue as potential collagen sources

**Figure 8:**
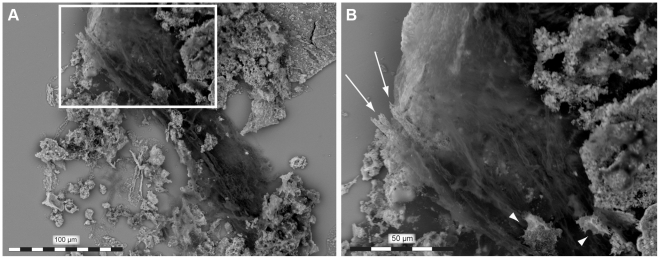
Partially mineralized fiber bundle obtained from IRSNB 1624. (A) Scanning electron micrograph of a partly mineralized fiber bundle located in between mineralized fragments of vessel-like structures. (B) Close up of the area marked in A showing partly mineralized fibers (arrows – note transition from mineralized to organic part of the fibers) and osteocyte-like entities (arrowheads).

Given that the mosasaur humerus was housed in the collections at IRSNB for many years, the possibility existed that the collagenous matter identified herein was non-authentic, and instead originated from fungal growth or gelatin-based bone glue. However, histological sections of untreated bone revealed that the fibrous microstructures were deeply embedded in hydroxyapatite prior to demineralization (Figure 1M). Moreover, TOF MS and DNA analyses failed to detect any ergosterol or nucleic acids attributable to fungi, and we were unable to identify any substances that could be related to bone glue (for instance, the vessel lumina were draped by iron oxide crystals, not organic matter; Figure 4). Likewise, the amount of finite carbon was exceedingly small, corresponding to 4.68%±0.1 of modern ^14^C activity (yielding an age of 24 600 BP), and most likely reflect bacterial activity near the outer surface of the bone (although no bacterial proteins or hopanoids were detected, one bacterial DNA sequence was amplified by PCR, and microscopic clusters of bone-boring cyanobacteria were seen in places along the perimeter of the diaphyseal cortex). Two short DNA sequences of possible lagomorph origin were amplified by PCR (together with three human sequences), and consequently it is possible that the outer surface of the bone has been painted with animal glue at some point. Nonetheless, based on the extremely weak PCR products obtained from the DNA analysis (8–26 ng/μl after two rounds of PCR and doubling up of the PCR reaction volume, suggesting very few copies of template DNA prior to PCR), the amount of lagomorph contamination is exceedingly small and cannot account for the relatively large quantities of fibrous matter located in between the vessel-like forms (i.e., in the area of the osteoid). Additionally, some fiber bundles are partially mineralized (Figure 8), providing convincing evidence for their antiquity. Accordingly, we find it reasonable to conclude that the collagenous biomolecules recovered from the fibrous tissues of IRSNB 1624 are primary.

## Discussion

Although IR spectroscopy by itself cannot generally be used to identify specific proteins, it can nonetheless provide invaluable information on the molecular content in samples of unknown composition [8], [9], [32]. Likewise, no other method employed herein stands alone (i.e., provides sufficient evidence for the survival of proteinaceous macromolecules over deep time); however, when combined the data obtained from our OM (Figure 1G, H), SEM (Figures 1A, B, 5A, and 8), TEM (Figure 1I, K), amino acid (Figure 2), antibody (Figure 3), IR (Figures 5–7), histochemical (Figure 1L), and histological (Figure 1M) analyses provide compelling evidence to suggest that primary organic molecules, including collagen or its degradation products, are preserved in the fibrous bone tissues of IRSNB 1624. Thus, under the appropriate conditions biomolecular preservation is not limited to large-sized bones buried in fluvial sandstone environments [6] but also occurs in relatively small skeletal elements deposited in shallow-marine sediments. For osteoid proteins, these conditions probably include protection from biodegradation by entombment in the highly resistant hydroxyapatite crystals of bone (Figure 1M) [33], which in IRSNB 1624 may have been facilitated by the small diameter (20–30 nm) of the fibrils (resulting in a high mineral-to-extrafibrillar ratio). Moreover, high levels of phosphate and carbonate in the Ciply Phosphatic Chalk might have minimized dissolution and re-precipitation of the bone mineral. This limited the exposure of the protein molecules to microorganisms, thus retarding the extent of microbial degradation. Also, because microorganisms can only infiltrate bones via cracks or natural cavities, early intravascular mineralizations may have blocked off internal surfaces (Figure 4), thereby denying bacteria access to organic material in the bone matrix [34].
